# Alternative Antimicrobial Approach: Nano-Antimicrobial Materials

**DOI:** 10.1155/2015/246012

**Published:** 2015-03-16

**Authors:** Nurit Beyth, Yael Houri-Haddad, Avi Domb, Wahid Khan, Ronen Hazan

**Affiliations:** ^1^Department of Prosthodontics, The Hebrew University-Hadassah School of Dental Medicine, P.O. Box 12272, 91120 Jerusalem, Israel; ^2^Department of Medicinal Chemistry, School of Pharmacy, Faculty of Medicine, The Hebrew University of Jerusalem, P.O. Box 12065, 91120 Jerusalem, Israel; ^3^Department of Pharmaceutics, National Institute of Pharmaceutical Education & Research (NIPER), Balanagar, Hyderabad 500 037, India; ^4^Institute of Dental Sciences, The Hebrew University-Hadassah School of Dental Medicine, P.O. Box 12272, 91120 Jerusalem, Israel; ^5^IYAR, The Israeli Institute for Advanced Research, Tel Aviv, Israel

## Abstract

Despite numerous existing potent antibiotics and other antimicrobial means, bacterial infections are still a major cause of morbidity and mortality. Moreover, the need to develop additional bactericidal means has significantly increased due to the growing concern regarding multidrug-resistant bacterial strains and biofilm associated infections. Consequently, attention has been especially devoted to new and emerging nanoparticle-based materials in the field of antimicrobial chemotherapy. The present review discusses the activities of nanoparticles as an antimicrobial means, their mode of action, nanoparticle effect on drug-resistant bacteria, and the risks attendant on their use as antibacterial agents. Factors contributing to nanoparticle performance in the clinical setting, their unique properties, and mechanism of action as antibacterial agents are discussed in detail.

## 1. Introduction

Bacterial infections are still a major cause of morbidity and mortality. The growing concern regarding multidrug-resistant bacterial strains and biofilm-associated infections calls for the development of additional bactericidal means. Consequently, attention has been especially devoted to new and emerging nanoparticle-based materials in the field of antimicrobial chemotherapy.

Bacteria are naturally found in clinical and industrial settings in association with surfaces. Although modern microbiological research focuses mainly on pure culture planktonic (free-swimming) bacteria, it is now generally recognized that most bacteria live in microbial communities, which are often composed of multiple species that interact with each other and their environment. Bacterial surface contamination, the adhesion, persistence, and colonization of surfaces by bacteria, is increasingly recognized as detrimental to health and society [1]. Biofilm-associated infectious diseases account for over 80 percent of microbial infections in the body, resulting in increased patient morbidity and medical expenses [2].

Biofilms are agglomerates of microorganisms that adhere to a substrate. First, the bacteria bind reversibly to the surface and then secrete binding molecules such as adhesion proteins that cause irreversible attachment. Once settled, the bacteria proliferate and form colonies inside peptidoglycan envelopes, which leads to the development of a mature biofilm. At this stage the bacteria not only become inaccessible to antibacterial agents and the body's immune system, but also provide a reservoir of bacteria for chronic infections throughout the body [3]. This is why biofilms are a severe health threat. Moreover, biofilms respond poorly to conventional antibiotics and may develop antibiotic resistance [2]. Thus, despite the numerous existing potent antibiotic drugs and other modern antibacterial means, bacterial infections are still a challenge.

Antimicrobial materials used in the clinical setting today are beset by significant shortfalls, including weak antimicrobial activities, risk of microbial resistance, difficulty in monitoring and extending the antimicrobial functions, and difficulty in functioning in a dynamic environment. Thus, effective and long-term antibacterial and biofilm-preventing materials constitute an immediate need in medicine and dentistry. Today, most biofilm-associated infections are treated with antibiotics for lack of a better alternative. However, it is well established that attacking mature biofilms with conventional antibiotics does not work; that is, much higher than usual drug doses are required, as all such agents have difficulty in penetrating the extracellular polysaccharide sheath covering the biofilm. Biofilm-associated bacteria are 100 to 1,000 times less susceptible to antibiotics than planktonic bacteria, and agents active against planktonic bacteria, but not against biofilms, fail to cure patients [4]. Moreover, high doses are often not tolerated by the host organism, whereas the conventionally used lower doses are inefficient. In addition, the use of conventional antibiotics carries a major risk for resistance of viable bacteria. This issue becomes more complicated in situations where mixed bacterial biofilms are produced and where multiple antibiotics are used to target the complex microflora. Consequently, different measures of antimicrobial protection are required. Nanotechnology today provides a sound platform for adjusting the physicochemical properties of numerous materials to generate effective antimicrobials [5]. Nanomaterials (NM) may be strategically advantageous as active antibacterial groups since their surface area is exceedingly large relative to their size. Nanosized particles may provide high activity although only a small dose of the particles is used. Consequently, NM could serve as an alternative to antibiotics to control bacterial infections.

The major groups of antibiotics, currently in use, generally affect three bacterial targets: cell wall synthesis, translational machinery, and DNA replication [6]. Unfortunately, bacterial resistance may develop against each one of these modes of action. Mechanisms of resistance include enzymes that modify or degrade the antibiotic such as *β*-lactamases and aminoglycosides, modification of cell components such as cell wall as seen in vancomycin resistance [6] and ribosomes in tetracyclines resistance, and finally efflux pumps that provide multidrug resistance against numerous antibiotics [6]. Since nanoparticles' mode of action is mainly by direct contact with the bacterial cell wall, without the need to penetrate the cells, most of the resistance mechanisms seen with antibiotics are irrelevant. This raises the hope that nanoparticles would be less prone than antibiotics to promote resistant bacteria.

In this review the potential of various NM as antimicrobial agents is described. The antibacterial mechanism of action of nanoparticles and their interactions with microbial cells leading to cell death, including a detailed discussion of toxic and biocompatibility properties, is provided.

## 2. Antimicrobial Nanoparticles

**Scheme 1:**
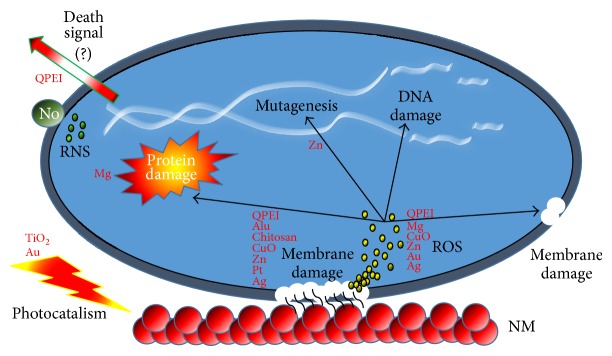
NM antibacterial mode of action. General schematic depicting the common modes of action of NM. Most known antibacterial NM interact electrostatically with the bacterial membrane causing membrane disruption. Frequently, free radicals (ROS yellow spots) are produced due to the NM-membrane interactions. These radicals may instigate secondary membrane damage, hinder protein function, cause DNA destruction, and result in excess radical production. Other antibacterial NM are photoactivated (photocatalism). Nitric oxide (NO) NM are involved with RNS (green spots). Polycationic NM (QPEI) have a unique feature as they seem to induce signal secretion that may promote programmed cell death.

Nanomaterials as antibacterials complementary to antibiotics are highly promising and are gaining large interest as they might fill the gaps where antibiotics frequently fail. This includes combatting multidrug-resistant mutants and biofilm [7, 8]. Antimicrobial NM now in use (i.e., metal, metal oxide, and organic nanoparticles) show a diversity of intrinsic and modified chemical composition properties. Thus, it is not surprising that they have numerous modes of action (Scheme 1). Furthermore, the target bacteria vary greatly in their genetics and consequently in their cell wall structure, essential metabolic pathways, and many components that when disrupted could be lethal to the microorganisms. Also, the physiological state of the bacteria, that is, planktonic, biofilm, growth rate, stationary, or starved, may greatly contribute to the sensitivity of the bacteria to NM [9, 10]. In some cases the ratio between the bacteria and the NM is critical to the latter's toxicity [11]. In addition, many environmental factors play a role and affect the lethality of NM to bacteria including aeration, pH, and temperature. The physicochemical properties of the particles including size, shape, chemical modification and coating, and mixture in various ratios with other nanoparticles and solvent used all affect greatly their antibacterial activity [12]. Thus, with this complexity, no wonder that large parts of the NM antibacterial mode of action and level of hazard they pose are still obscure and one can find in the literature contradictory reports about them [13, 14].

Nevertheless, in general, NM act along two major lethal pathways, which are related to each other and in many cases occur simultaneously: (1) disruption of membrane potential and integrity and (2) production of reactive oxygen species (ROS), also known as oxygen-free radicals, the NM acting as nanocatalysts [7, 11, 15].

Membrane damage occurs when NM bind electrostatically to the bacterial cell wall and membranes, leading to alteration of membrane potential, membrane depolarization, and loss of integrity which, in turn, result in an imbalance of transport, impaired respiration, interruption of energy transduction and/or cell lysis, and eventually cell death [7]. ROS, considered the most effective determinant for both the* in vitro* and* in vivo* cytotoxicity of NM, are induced indirectly due to respiratory chain disruption or directly by the NM themselves [16]. A burst of ROS causes, via severe oxidative stress, damage to all the cell's macromolecules, leading to lipid peroxidation, alteration of proteins, inhibition of enzymes, and RNA and DNA damage. At high concentrations the ROS lead to cell death and at low doses cause severe DNA damage and mutations [17, 18]. In some cases, where the production of ROS is induced by visible or UV light [19] the toxicity of NM is photocatalytic. For instance, TiO_2_ NM were shown to induce, under near-UV light, lipid peroxidation which leads to respiratory dysfunction and death of* E. coli* cells [20].

Several other effects of NM include direct inhibition of specific essential enzymes, induction of nitrogen reactive species (NRS) [7, 11, 14, 15], and induction of programmed cell death [21].

## 3. Inorganic Nanoparticles

*Metals and metal oxides* have been widely studied for their antimicrobial activities [22]. Metal oxide nanoparticles, well known for their highly potent antibacterial effect, include silver (Ag), iron oxide (Fe_3_O_4_), titanium oxide (TiO_2_), copper oxide (CuO), and zinc oxide (ZnO). Most metal oxide nanoparticles exhibit bactericidal properties through reactive oxygen species (ROS) generation although some are effective due to their physical structure and metal ion release. Representative synthesis/preparation of selected antimicrobial NM is shown in Table 1.

### 3.1. Silver

Of the metal nanoparticles, silver nanoparticles have been widely used as an effective antimicrobial agent against bacteria, fungi, and viruses [23]. Their effect was recognized already in ancient times. Ag and its compounds have long been used for the disinfection of medical devices and water purification. In medicine, Ag compounds are commonly applied to treat burns, wounds, and a variety of infectious diseases [24–26]. The antimicrobial efficacy of Ag, as of other metals and metal oxide nanoparticles, was reported to be size-dependent [27]. Although the Ag nanoparticle mechanism of action is still not clear, small diameter Ag nanoparticles have a superior antimicrobial effect to those of a larger diameter [28]. Moreover, Ag nanoparticle antibacterial activity exceeds that of their bulk equivalents. Nonetheless, high surface energy may compromise their efficacy due to their susceptibility to aggregate into large particles, which may result in the loss of their antibacterial activity.

Silver (Ag), similarly to other nonantibiotic treatments, was almost abandoned when penicillin and later on other antibiotics were discovered. But today, with the emergence of antibiotic-resistant strains, it has gained new, yet controversial, interest [29]. Silver was reported to be an efficient bactericidal antibacterial agent against various pathogens* in vitro* and* in vivo* [30]. Moreover, it seems that bacteria are less prone to develop resistance against Ag than against conventional antibiotics [31, 32]. However, several points of controversy remain to be resolved: the debate and questions on the definition and determination of silver minimal inhibitory concentration (MIC) and breaking point, the ease of emergence of resistant strains [33, 34], whether silver really kills biofilm or just planktonic cells [35], and the side effects of Ag on humans [36–38]. In addition, the bactericidal mechanisms of Ag-NM are not fully understood [39]. In* E. coli*, as a representative of Gram-negative bacteria, Ag nanoparticles were shown to cause “pits” in the cell wall by increasing the membrane permeability and inactivating the respiratory chain [21, 40]. Other investigations showed that the Ag ion, which has an affinity for sulfur and nitrogen, can inhibit and disrupt protein structure by binding to thiol and amino groups [41]. Finally it was suggested that silver NM are photocatalytic [42] and can induce ROS [43–45], an observation that was contradicted by others showing that, at least in eukaryotic cells, this effect is cell-type dependent [46, 47]. Ag-NM were shown also to have synergistic antibacterial effects both on Gram-positive and Gram-negative bacteria when provided in combination with antibiotics [48, 49]. However, despite the controversies and ongoing debates, Ag-NM are perhaps the most promising antibacterial metal NM.

### 3.2. Titanium Oxide

Titanium dioxide (TiO_2_) is another metal oxide that has been extensively studied for its antimicrobial activities [50]. TiO_2_ has long been known for its ability to kill both Gram-positive and Gram-negative bacteria [51]. More recent reports have shown its efficiency against various viral species and parasites [52–54].

Titanium dioxide (TiO_2_) NM as antibacterial compounds have been on the market for quite some time [20]. Similar to Au, they are photocatalytic; their toxicity, induced by visible light, near-UV or UV [7], stimulates ROS burst. The ROS damage the membrane, DNA, and many other macromolecules and functions of the bacterial cell [15]. TiO_2_ is effective against many bacteria including spores of* Bacillus* [55], which is the most resistant organism known. As with other NM, combinations of Ti or TiO_2_ with other NM such as Ag were found to have a synergistic effect and to enhance their activity [56–58].

### 3.3. Zinc Oxide

Additional broad spectrum bactericidal NM are ZnO-based nanoparticles [59]. ZnO nanoparticles were shown to have a wide range of antimicrobial activity against various microorganisms, which is significantly dependent on the chosen concentration and particle size [59]. Moreover, ZnO nanoparticles were shown to inhibit the growth of methicillin-sensitive* S. aureus* (MSSA), methicillin-resistant* S. aureus* (MRSA), and methicillin-resistant* S. epidermidis* (MRSE) strains and proved to be effective bactericidal agents that were not affected by the drug-resistant mechanisms of MRSA and MRSE [60, 61]. Zinc oxide (ZnO) NM are of relatively low cost [11] and effective in size dependency [59] against a wide range of bacteria [62, 63]. These include pathogens such as* Klebsiella pneumonia* [64],* Listeria monocytogenes*,* Salmonella enteritidis* [65],* Streptococcus mutans, Lactobacillus* [66], and* E. coli* [65, 67] with low toxicity to human cells [68]. Their white color, UV-blocking, and ability to prevent biofilm formation makes them suitable for fabric [69] and glass [70] industries as coating materials designated for medical and other devices. Furthermore, treatment using zinc was approved by the FDA and nowadays Zn is available as a food additive [15].

ZnO NM affect bacterial cells along the two pathways, described above, by binding to membranes, disrupting their potential and integrity, and by inducting ROS production [65, 67, 71]. In addition and as a result, Zn NM are also mutagens, albeit weak ones [17].

### 3.4. Iron Oxide and Gold

Fe_3_O_4_ nanoparticles and gold (Au) represent an additional class of antimicrobial materials that are being researched for their use in health care [72]. Fe_3_O_4_ in its bulk form and Au are generally considered inert and lack antimicrobial properties. Interestingly, these materials can be modified to introduce antimicrobial properties when synthesized as nanosize particles. Microbiological assays have proved that surfaces modified using Fe_3_O_4_ nanoparticles demonstrate antiadherent properties and significantly reduce both Gram-negative and Gram-positive bacterial colonization [73]. Au nanoparticles and nanorods have been reported to be bactericidal when photothermally functionalized [74].

In comparison to Ag, gold- (Au-) NM are less potent and have almost no antibacterial effect by themselves [39]. Nevertheless, Au-NM bound to antibiotics such as ampicillin [75, 76], vancomycin [77], the antibacterial enzyme lysozyme [78], and even other NM [79] were bactericidal to many multidrug-resistant pathogens, including those which were penicillin and vancomycin resistant. Au-NM antibacterial activity was enhanced also by binding to nonantibiotic molecules such as amino-substituted pyrimidines [80] and citrate, which together with light energy, induced ROS production and mutations used in therapy against cancer cells [81]. Another example of an antibacterial approach, adopted from cancer treatments, is the Au-NM bound to Fe_3_O_4_ and activated by photothermal treatment [82]. The stability of Au-NM compared with that of other metal NM, such as platinum (Pt) [83], render them in many cases the preferred antibacterial NM.

Most of the knowledge about Pt NM comes from cancer research where it was shown in mammalian cells that Pt NM diffuse through membranes and induce DNA damage, accumulation of cells at the S-phase of the cell cycle, and apoptosis [84]. Recently, however, the toxicity of Pt NM to bacteria was also demonstrated and found to be size-dependent. Pt NM particles of 1–3 nm size were bactericidal to* P. aeruginosa* cells, whereas those of 4–21 nm size exhibited bacteriocompatible properties [85].

Another recent study showed that when Pt and Au, each alone nontoxic to bacteria, are combined in a bimetallic setting, they have a strong bactericidal effect [86]. Interestingly, in contrast to other NM this effect was ROS-independent, cell death resulting according to the authors from membrane damage and a severe elevation of ATP [86].

### 3.5. Copper Oxide

Although copper oxide (CuO) nanoparticles have been shown to be effective against various bacterial pathogens, their antibacterial efficacy is somewhat inferior to that of Ag or ZnO. Hence, a comparatively higher concentration of nanoparticles is needed to achieve the same results [87]. Moreover, CuO nanoparticle activity varies greatly depending on the challenged bacterial species. Nonetheless, as Cu is much less expensive than other nanosized metal materials, it can be utilized for efficacy enhancement in the form of nanocomposites.

Copper oxide (CuO) NM, like the other metallic nanoparticles, exert their antibacterial activity [88, 89] by membrane disruption and ROS production [7]. In general, Co NM are less potent than Ag-NM, although in some cases the reverse is true. For example,* E. coli* and* S. aureus* were more sensitive to silver, whereas* B. subtilis* and* B. anthracis* were more sensitive to Cu NM [90, 91]. A comparison of CuO NM with metallic MN other than Ag-NM showed that they have the strongest antibacterial activity [9, 92]. A possible explanation for these observations is that bacteria, such as* B. subtilis*, with cell walls rich in amine and carboxyl groups, bind more strongly to CuO and thus are more sensitive to it [7, 11, 15]. Thus it seems that in special cases it would be beneficial to use the CuO NM instead of others, including silver.

### 3.6. Magnesium Oxide

Nano-magnesium oxides (MgO) are additional antibacterial metal oxide NM that have been shown to exhibit bactericidal activity. Nano-MgO particles were reported to exhibit efficient antimicrobial activity against bacteria (both Gram-positive and Gram-negative), spores, and viruses. Compared to other metal nanoparticles, nano-MgO has the advantage that it can be prepared from available and economical precursors.

Magnesium (Mg) can be used in various NM in the form of MgO or MgX_2_ (e.g., MgF_2_) [7, 93]. In addition to inducing ROS, Mg-containing NM may directly inhibit essential enzymes of the bacteria [15]. MgF_2_ NM were found to prevent biofilm formation of* E. coli* and* S. aureus* [94, 95].

### 3.7. Superparamagnetic Iron Oxide

Superparamagnetic iron oxide (SPION) represents a relatively new approach using magnetic particles that cause local hyperthermia in the presence of a magnetic field [96] or, alternatively, they can be coated by other NM such as Ag and Au and their magnetic effect can be utilized to penetrate and destroy biofilms [14, 97–99].

### 3.8. Nitric Oxide

Nitric oxide (NO) NM presents a promising antibacterial compound due to the low risk of possible resistance; that is, NO is involved in multiple mechanisms of antimicrobial activity [100, 101]. As other metal-based nanoparticles the antibacterial effect is dependent on size and shape [102]; the smaller particles with a high aspect ratio are the most effective. NO is an endogenously produced molecule which is involved in various physiologic functions. Despite all its advantages, its clinical value is limited mainly because it is extremely reactive. However, NO's antimicrobial potential can be exploited upon its encapsulation, controlled release, and focal delivery [103].

Nitric oxide (NO) NM differ from other metal NM by specifically affecting reactive nitrogen species (RNS), rather than ROS. NO NM were found to effectively kill methicillin-resistant* S. aureus* (MRSA) [104] in skin infections [105] and to enhance wound healing of normal and diabetic mice [106]. NO NM are also effective in biofilm eradication of multiple bacterial species [107–109].

### 3.9. Aluminum Oxide

It is not clear if aluminum oxide (Al_2_O_3_) nanoparticles are suitable for antibacterial treatment. First, their bactericidal effect is relatively mild and they work only at high concentrations [7, 110] unless in combination with other NM such as Ag [111]. Second and more disturbing is their ability to promote horizontal transfer of multiresistance genes mediated by plasmids across genera [110].

The mechanism of action of aluminum NM, as recently shown for* E. coli*, is by diffusion and accumulation inside the cells, causing pit formation, perforation, and membrane disorganization, leading to cell death [112].

## 4. Organic Nanoparticles

Polymeric nanoparticles kill microorganisms either by releasing antibiotics, antimicrobial peptides, and antimicrobial agents or by contact-killing cationic surfaces such as quaternary ammonium compounds, alkyl pyridiniums, or quaternary phosphonium. Multiple mechanisms of action have been proposed for how these cationic groups are able to disrupt the bacterial cell membrane, with some requiring hydrophobic chains of certain lengths to penetrate and burst the bacterial membrane. It has been shown that high levels of positive charge are capable of conferring antimicrobial properties irrespective of hydrophobic chain length, perhaps by an ion exchange mechanism between the bacterial membrane and the charged surface. The antibacterial effect of polycations is dependent on the ability of multiple charges to attach to and interact with the cell membrane. These findings suggest the possibility of engineering a variety of polymer based positively charged surfaces to create a wide range of contact-killing materials [113].

Organic antibacterial materials are considered less stable in nature mainly at higher temperature when compared with inorganic materials. This may lead to difficulties that arise when designing products meant to be stable and able to withstand harsh process conditions. Therefore inorganic nanosized materials have been more often used as antimicrobial materials. A comprehensive review on antimicrobial polymers has been published [114]. A brief summary of the polymers mentioned in this review is given below.

### 4.1. Poly-*ɛ*-lysine

Poly-*ɛ*-lysine is a cationic homopeptide of L-lysine which is effective against Gram-positive and Gram-negative bacteria. It also displays activity against spores of* B. coagulans, B. stearothermophilus*, and* B. subtilis* [115].

### 4.2. Quaternary Ammonium Compounds

Quaternary ammonium compounds such as benzalkonium chloride, stearalkonium chloride, and cetrimonium chloride are well known disinfectants. Their antimicrobial activity is a function of the N-alkyl chain length and hence lipophilicity. Compounds with alkyl chain length 12–14 of alkyls provide optimum antibacterial activity against Gram-positive bacteria, while alkyls group with 14–16 carbon chains show better activity against Gram-negative bacteria. Initial interaction with bacterial wall results from electrostatic interaction between positively charged moieties of the compound and negatively charged bacterial membranes, followed by the integration of the hydrophobic tail of the compound into the bacterial hydrophobic membrane core, where they denature structural proteins and enzymes.

Antimicrobial polymers with only one biocide end group on polymeric backbone were synthesized by cationic ring-opening polymerization of 2-alkyl-1,3-oxazolines, terminating the macromolecule with a cationic surfactant [116]. Quaternary pyridiniums are compounds with a heterocyclic ring containing nitrogen atom. The antibacterial activity is a function of the pyridinium group in the polymer chain. Another family of antimicrobial polymer with aromatic/heterocyclic groups is imidazole derivatives. Imidazole possesses the ability to form hydrogen bond with drugs and proteins while its alkylated form (imidazolium) has the ability to aggregate electrostatically despite losing the hydrogen bond-forming ability of free imidazole. They are chemically stable and biocompatible and show improved biodegradability [117]. Copolymers of N-vinylimidazole and phenacyl methacrylate were synthesized; they display strong antimicrobial activity against various bacteria, fungi, and yeast [118]. Polyethyleneimine (PEI) is a synthetic, nonbiodegradable, cationic polymer containing primary, secondary, and tertiary amino functions. PEI was attached to various organic and inorganic, natural and synthetic, macroscopic and nanoscaled, monolithic, and porous surface materials including commercial plastics, textiles, and glass. These immobilized surfaces resulted in inactivation of both waterborne and airborne bacteria and fungi, including pathogenic and antibiotic-resistant strains without any report of emergence of resistance. Cell membrane rupture was reported as a main mechanism for antibacterial action. These surfaces are nontoxic for mammalian cells. N-alkylated PEIs immobilized over different woven textiles (cotton, wool, and polyester) also exhibit strong bactericidal activity against several airborne Gram-positive and Gram-negative bacteria. Mw of PEI poses a significant effect on activity. Substituted PEIs were also used against* Candida albicans,* presenting a major challenge for the safety of prosthesis deterioration in laryngectomized patients. Polyguanidines and polybiguanides represent an important class of antimicrobial polymers because of their high water solubility, excellent biocidal efficiency, wide antimicrobial spectrum, and nontoxicity. Acrylate monomers with pendant biguanide groups display good antimicrobial action due to electrostatic interaction with cell membranes. A series of different oligomeric guanidines by polycondensation of guanidinium salts and four different diamines under various conditions have been synthesized. The compounds of these series are linear in structure and can be recognized by termination with one guanidine and one amino group (type A), two amino groups (type B), or two guanidine groups (type C), respectively. An average molecular mass of about 800 Da is necessary for efficient antimicrobial activity [119].

### 4.3. Cationic Quaternary Polyelectrolytes

Most of the known cationic quaternary polyelectrolytes employed as antimicrobial polymers are acrylic or methacrylic derivatives, and a large number of them are synthesized from commercial methacrylic monomers such as 2-(dimethylamino)ethyl methacrylate. These polymers provide wide structural versatility by the alteration of hydrophobicity, molecular weight, surface charge, and other parameters [120].

### 4.4. N-Halamine Compounds

N-halamine compounds contain one or more nitrogen-halogen covalent bonds that are usually formed by halogenation of imide, amide, or amine groups, which provide stability and slow release free active halogen species into the environment. These oxidizing halogens promote the direct transfer of an active element to the biological target site or through dissociation to free halogen in aqueous media. These reactive free halogens lead to inhibition or inactivation of a microbial cell [121].

### 4.5. Polysiloxanes

Another important class of polymers is polysiloxanes, the linear polymers of silicon oxide. Sauvet et al. synthesized statistical and block siloxane copolymers containing quaternary ammonium salt groups as a lateral substituent; this research shows high antibacterial activity against both* Escherichia coli* and* Staphylococcus aureus*. However, no difference in activity was observed in block type polymers and statistical copolymers [122].

### 4.6. Benzoic Acid, Phenol, and p-Hydroxy Benzoate Esters

Benzoic acid, phenol, and p-hydroxy benzoate esters are among the most widely used disinfectants and preservatives. As monomers these compounds have already established their antimicrobial activity. Attempts have been made to incorporate them with some polymer backbone to synthesize new antimicrobial polymers with enhanced activity. In a comparative study of p-hydroxyphenyl acrylate, allyl p-hydroxyphenyl acetate, and* p*-2-propen oxyphenol for their antimicrobial action against both bacteria and fungi, p-hydroxyphenyl acrylate has been shown to be the most effective [123]. The stereo electronic effect of the phenyl group is a major contributing factor for antimicrobial activity of p-hydroxyphenyl acrylate derivatives. Compounds with acryl or acryloxy groups bound to the phenyl moiety exhibit better antimicrobial activities than aliphatic acrylates and hexyl acrylate [124]. Another important compound of this class is “benzaldehyde,” known for its bactericidal, fungicidal, and algaecidal activities. Benzaldehyde containing methyl methacrylate polymers have been synthesized and tested against* Bacillus macroides, Pseudomonas aeruginosa*, and* Dunaliella tertiolecta*. Polymers show fivefold inhibition of algae growth compared to acid-glass control surfaces [125].

### 4.7. Quaternary Phosphonium or Sulfonium Groups

Polymers possessing quaternary phosphonium or sulfonium groups display mechanisms similar to the quaternary ammonium group containing compounds. In terms of antimicrobial activity, phosphonium containing polycationic biocides are more effective than quaternary ammonium salt polymers. Studies carried out on water soluble thermosensitive copolymer NIPAAm and methacryloyloxyethyl trialkyl phosphonium chlorides indicate that the antimicrobial activity increases with an increase in length of the alkyl chain and phosphonium units in the polymer [126].

### 4.8. Triclosan

One of the most widely used antimicrobial agents is triclosan. In experiments solutions of triclosan were mixed with water-based styrene-acrylate emulsion; the resultant systems were tested against* Enterococcus faecalis*. Based upon an agar diffusion test, it was demonstrated that the release of triclosan depends on the solvent, being almost inexistent or very slow with water and very rapid with n-heptane [127]. In another experiment triclosan was incorporated in water-dispersible PVA nanoparticles that shows greater antibacterial activity towards* Corynebacterium* than the organic/aqueous solutions of triclosan [128].

### 4.9. 5-Chloro-8-hydroxy-quinoline

Acrylate polymers containing 5-chloro-8-hydroxy-quinoline were studied at physiological, acidic, and basic pH for their hydrolytic behavior. Hydrolysis occurs by autocatalysis and is potentiated by pH, temperature, and the content of hydrophilic polymers. Copolymerization of this polymer with N-vinyl pyrrolidone reduces the rate of hydrolysis due to steric hindrance [129].

### 4.10. Peptides

Various peptides were synthesized via ring-opening polymerization of *α*-amino acid N-carboxyanhydride (NCA) monomers using lysine (K) as the hydrophilic amino acid and alanine (A), phenylalanine (F), and leucine (L) as hydrophobic amino acids. They varied the content of hydrophobic from 0 to 100% and obtained five series of copeptides (i.e., P(KA), P(KL), P(KF), P(KAL), and P(KFL)). MIC values determination against* Escherichia coli*,* Pseudomonas aeruginosa*,* Serratia marcescens*, and* Candida albicans* demonstrate that the P(KF) copeptides have broader antimicrobial activity and are more efficient than the P(KL) and P(KA) series. Similarly, the P(KFL) series is more effective than the P(KAL) series [130].

### 4.11. Organometallic Polymers

Organometallic polymers contain metals either in the backbone chain or in the pendant group, bonded to the polymer by Π-bonds to carbon, coordination bonds to elements containing free electron pairs, or *ρ*/Π-bonds to other elements. Carraher et al. synthesized organotin polyamine ethers containing acyclovir in their backbone. Many such compounds were synthesized by varying alkyl group (methyl, ethyl, butyl, octyl, cyclohexyl, and phenyl) and tested against herpes simplex virus-1 (HSV-1) and Varicella zoster virus (VZV). These polymers present a good inhibition of both RNA and DNA viruses [131].

### 4.12. Polymeric Nanosized Antimicrobials

Polymeric nanosized antimicrobial agents are known to have long-term antimicrobial activity: they are nonvolatile and chemically stable, can bind to the surface of interest, and hardly permeate through biological membranes such as the skin [132]. Distinctively, polycationic antimicrobials have a high surface density of active groups which might result in increased antimicrobial activity. Quaternary ammonium compounds have a broad spectrum of antimicrobial activity against both Gram-positive and Gram-negative bacteria. Polyamines that have been reported as being highly effective antimicrobial nanoparticles are quaternary ammonium polyethylenimines (QPEI), which have a broad range of bacterial targets when incorporated in various polymeric matrixes [133, 134]. Similarly, lipid nanoparticles are attractive for their biocompatibility, versatility, and their ability to target biofilm infections.

### 4.13. Polycationic Nanoparticles

QPEI are unique among other NM in their ability to induce intracellular death signal. This yet unidentified signal causes death of cells in layers of biofilm that are not in direct contact with the nanoparticles [21]. This observation, that NM might induce bacterial programmed cell death, is extremely interesting. Such signals, if identified, may theoretically be used to enhance the NM's activity and efficacy. Moreover, such signals may efficiently be the answer to one of the principal shortfalls of antibiotics, being their poor ability to penetrate biofilms. The field of programmed cell death (PCD) in bacteria is still enigmatic and controversial, yet there is growing evidence that PCD plays an important role in the life cycle of bacterial cultures and moreover that it is regulated by secreted signals.

### 4.14. Chitosan

Chitosan (Ch) nanoparticles have also been shown to have broad spectrum antibacterial, antiviral, and antifungal activity. Lately, Chitosan-hydroxycinnamic acid conjugates were introduced with high bactericidal activity [135]. The widespread applications of Ch are primarily based on their biocompatibility, nontoxic nature, antibacterial properties, low immunogenicity, and the ability to act as an absorption enhancer. Chitosan NM are nanoparticles obtained by N-deacetylation of the N-acetylglucosamine polymer chitin commonly found in the exoskeleton of insects. Chitosan nanoparticles display considerable antibacterial activity [136], which depends on several factors, including pH and solvent [137, 138]. Interestingly, chitosan reduced the activity of metal NM such as Zn [137]. Thus it appears that it should not be combined with metal NM but possibly with antibiotics [139].

The antibacterial mode of action of chitosan is not fully understood [11]. A recent comprehensive study of the effect of chitosan on* B. cenocepacia* indicated that many membrane-related functions were affected including respiration and resistance nodulation cell division (RND), drug efflux system, and transport. This is possibly due to interaction of lipopolysaccharides with chitosan, resulting in the destabilization of membrane protein sand membrane lysis, leading to cell death [140].

In summary, it seems that regarding NM's mode of action a lot is still obscure. Several NM killing pathways are still elusive and need to be discovered. The effects of NM's treatment combinations are still poorly understood. Last, the involvement of, yet controversial, bacterial intrinsic pathways of programmed cell death in NM's dependent killing needs to be further clarified.

## 5. Synthesis/Preparation Methods

Nano-antimicrobial materials can be synthesized by variety of different methods. Recent work showed that the mechanism of action and activity of materials may influence subsequent antimicrobial effect. Table 1 represents synthesis/preparation method for selected antimicrobial NM with material description and antimicrobial activity.

## 6. Biocompatibility of Nanomaterials

The biocompatibility of nanomaterials must be explored prior to their use in biomedical applications such as drug delivery, gene delivery, biosensors, or the treatment of wound infections. In such applications, the NM come in direct contact with tissues and cells, where they can cause beneficial or destructive effects on the body. NM as drugs can gain access to the body by inhalation, oral ingestion, intravenous injection, and contact with the skin [113]. The effect of NP on various body tissues is not known, and the interaction of NM with cells and tissues is poorly understood.

**Scheme 2:**
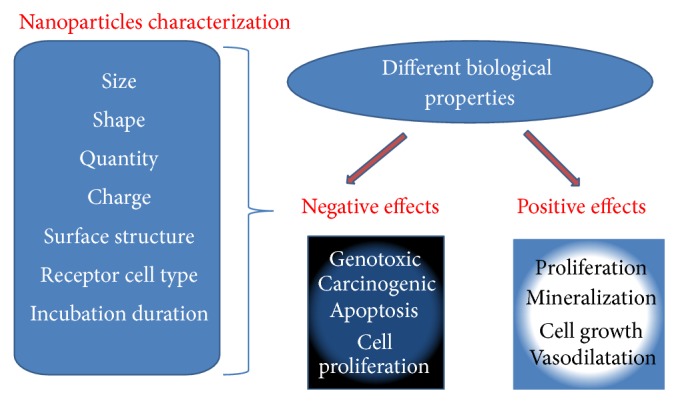
NM biocompatibility from* in vitro* studies. The biological activity of different organic and inorganic NM varies from negative to positive effects in different systems of* in vitro* cell lines. This activity depends on various factors such as size, electrical charge, quantity exposed, shape, and surface structure of NM.

The toxicity of NP can be assessed by a number of* in vitro* and* in vivo* methods (Scheme 2). The* in vitro* research is conducted on cell cultures. Cell culture assays are used as a prescreening tool to understand the biological effects of NM activity, their toxicity, and mechanism of action. A few inorganic and many synthesized polymeric NM have been shown to have different levels of biocompatibility. Herein, several such NM and their effective roles are discussed.

## 7. Inorganic Nanoparticles

Metal NM have in several studies been shown to be cytotoxic [114, 141], genotoxic [142], and potentially carcinogenic [143] and to induce apoptosis and inhibit cell proliferation [144]. Some studies found that NM toxicity depends on particle size and charges. Negatively charged 10 nm SiO_2_^−^ have a strong impact on cell viability and genotoxic effects, but the largest particles (100 nm) do not affect cell activity.

Ag-NP and Au-NM showed the best results in terms of toxicity and were defined as nontoxic for human cells [145].

Pure Ti and TiO_2_ are extensively used for dental and orthopedic implants owing to their high mechanical properties and biocompatibility. The biocompatibility of Ti is dependent on the characteristics of vertically aligned TiO_2_ nanoporous surfaces [146]. Titanium foils are covered by the vertically aligned nanoporous surface of TiO_2_, and the TiO_2_ nanoporous surface enhances the proliferation and mineralization of osteoblasts and increases mobility, as well as vasodilation of endothelial cells [147]. Giavaresi et al. found that nanostructured TiO_2_ coating had a positive effect on cell proliferation and activity [148]. Another study reported that the growth rate of osteoblast cells increased three- to fourfold in response to treatment with TiO_2_ nanotubes [149]. As mentioned above, some of the inorganic NM have toxic effects on both microbial and animal cells, and their relative biocompatibility and toxicity are dose- and cell-type dependent. Furthermore, with modification of their structure, effective levels of biocompatible properties have been observed in metals such as Ag [150].

A number of studies have reported the nontoxic and biocompatible behavior of SPION* nanoparticles* in different human and animal cells. Jian et al. investigated the* in vivo* behavior of SPION in rat liver and concluded that it did not influence liver function or induce oxidative stress [151]. Furthermore, Sun et al. showed good biocompatibility of sodium oleate-coated iron oxide NM [152].

Although the results of the cell culture studies are promising, the* in vitro* assays should be confirmed by* in vivo* studies conducted in animal models before NM applications are available for human use. The relatively small number of animal investigations designed to determine the toxicity of NM of different sizes and shapes, as well as dose-dependence, has not allowed conclusions to be drawn as to whether NM as potential antimicrobial agents are safe for humans. Therefore, NM toxicity studies are necessary to determine risk assessment.

Using ZnO nanowires (NWs) in Hela and L929 culture cells, Li et al. reported that Hela cells showed full biocompatibility with ZnO nanowires (NWs) at all concentrations. However, the multiplication capacity of L929 cells was good at lower NW levels whereas cell viability was reduced by 50% at higher levels of ZnO NW [153].

The cytotoxic behavior of nanomaterials is somewhat different in higher animal cells but still exists. Some NM, such as Ag, ZnO, and TiO_2_, show moderate to high levels of cytotoxicity against a variety of animal cells. In addition, some NM including SiO_2_, Au, Fe_2_O_3_, and TiO_2_ have also shown a very good level of biocompatible properties. Even cytotoxic NM have been converted into biocompatible materials through slight variations in their surface structure. Therefore, it may be concluded that NM possess a broad level of biological properties that are highly dependent upon their size, structure, quantity, and receptor cell type. However, further studies are still required to identify additional reasons for their behavior.

Moreover, the* in vivo* toxicological effects of NM are much more severe than their* in vitro* effects. Nanomaterials that penetrate the body through the skin, by respiration or by inhalation, directly affect major body organs including the lungs, heart, and brain.

The toxicity of Au-NM (4-5 nm) after rat inhalation was represented by a dose-dependent accumulation of gold in lungs, inflammation, and an increased number of macrophages [154]. de Jong et al. determined the size-dependent organ distribution of Au-NM (10, 50, 100, and 250 nm) after intravenous administration to rats. Their results showed that 10 nm Au-NM was the amount most widespread in the various organ systems, including brain, heart, kidneys, lungs, testis, and thymus. Oral toxicity, eye irritation, corrosion, and dermal toxicity of colloidal Ag-NM were conducted in mice and guinea pig models [155]. Their findings suggest that Ag-NM could be relatively safe if administered for short periods of time.

However, the exact toxicological mechanism of NM and the level of hazard they pose are unknown. The toxic effects of NM may be attributed to various factors. However, generation of ROS is considered the main determinant for both their* in vitro* and* in vivo* cytotoxicity. ROS is physiologically essential but potentially destructive to eukaryotic cells. Several cellular events are governed by lower levels of ROS, but when they increase beyond certain limits they cause severe oxidative stress, resulting in cell death via oxidation of the lipids and alteration of the DNA and proteins [156–158]. ROS generated from TiO_2_ NM caused oxidative stress that resulted in early inflammatory responses in mice, rats, and hamsters [159]. Oxidative stress has been shown to be generated by CNT in fish brain and to cause pulmonary inflammation in rats [160, 161]. The excessive generation of ROS has also been reported to damage mitochondrial DNA [162, 163]. The toxic effects generated by ROS are not confined to particular cells or organs but also affect various body systems and functions, including the central nervous system (CNS), respiratory system, and cardiac conduction [164, 165].

## 8. Organic Nanoparticles

Incorporation of QPEI-based nanoparticles at low concentrations did not change the biocompatibility results when compared with the commercial dental restorative materials. This effect was tested by cell viability (XTT) and TNF*α* secretion of monocytes challenged by these NM [166]. This biocompatibility of QPEI was also shown when the nanoparticles were incorporated in endodontic sealers [167] and soft liner materials.

It may be concluded that most NM have both cytotoxic and compatible properties. Moreover, these properties are highly dependent on various parameters, including the size of the NM, dose, cell type, and incubation duration. The properties can be customized by slightly modifying the surface or charge properties of the nanomaterial. However, a great deal of intensive research is still required to determine the basis for the various NM properties.

**Scheme 3:**
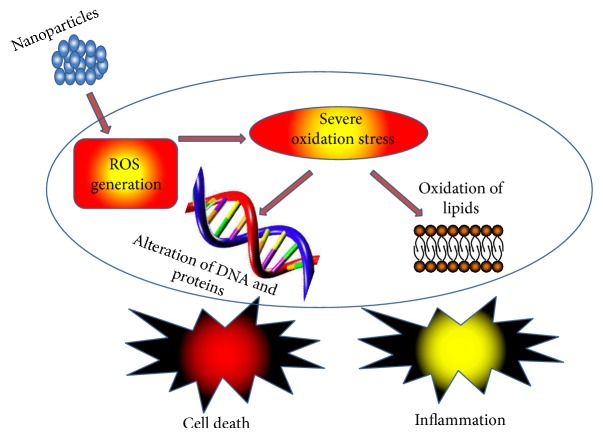
Toxicological mechanisms of NM to eukaryotic cells. Nanoparticles induce ROS generation in eukaryotic cells; these radicals cause severe oxidation stress in the cells, affecting membrane lipids and altering the structure of DNA and proteins. This excess radical production induces an inflammatory process that could lead to cell death.

Despite the numerous advantages that antibacterial NM offer, they also have some imperative shortcomings. Nanomaterials may be toxic to human cells and tissue, causing oxidative stress, disturbing enzymes activity, and causing membrane and DNA damage, all of which lead to cell death (Scheme 3). Nonetheless, recent studies show that NM have the potential to be efficient antibacterial agents, provided their main disadvantage, toxicity, will be addressed.

## 9. Summary

Bacterial strains resistant to the antibiotics now in use have become a serious public health problem that increases the need to develop new bactericidal materials. Consequently, there is a strong demand for developing novel strategies and new materials that can cope with these serious issues. The emergence of nanotechnology has created many new antimicrobial options. The small size of the NM is very suitable for carrying out antimicrobial biological operations. Metal, organic, and additional nanoparticle types have shown tremendous potential as bactericidal and fungicidal elements, demonstrating their potential as efficient antibiotic reagents in wound care and related medical issues. The efficacy of these nanoparticles varies with their characteristics including size, shape, and concentration. Moreover, the atomic abundance on the particles' surface plays a role in the properties of such materials. As the size of the particle decreases, the percentage of atoms on the surface increases relative to the total atoms of material, amplifying the activity. Various NM display antimicrobial activity against numerous pathogenic viral and bacterial species. Likewise NM have shown sufficient biocompatibility when incorporated in scaffold materials. Nanomaterials today are a promising platform for alternative measures to control bacterial infections.

Antimicrobial NM offers a wide range of classes and applications. These antimicrobial NM offer prolonged antimicrobial activity with negligible toxicity, compared with small molecular antimicrobial agents that display short-term activity and environmental toxicity. The emergence of resistant species is one of the major problems with small molecular antibiotics due to their specific targets of action, whereas antimicrobial NM physically destroys cell membranes of the organism which prevent development of drug-resistance microbes. Due to these advantages provided by antimicrobial NM, efforts have been made to apply these NM as contact surfaces for medical devices, fibers, and textiles, rendering them antimicrobial. Advanced quality research, dedicated efforts, successful application, and commercialization of antimicrobial NM will help fulfill the need to improve the quality of life.

## Figures and Tables

**Table 1:** Representative synthesis/preparation method for selected antimicrobial nanomaterials.

Material	Nanomaterial/particles description	Representative synthesis/preparation method	Reference
Titanium oxide (TiO_2_)	Nanosilver-decorated titanium dioxide (TiO_2_) nanofibers with antimicrobial activity were synthesized which displayed a self-cleaning property and toxic decomposition potential	Titanium nanofibers were prepared by electrospinning. Briefly, pluronic and PVP were each dissolved in ethanol. A TiO_2_ solution was prepared by adding titanium isopropoxide (TiP) in a mixture of ethanol and HCl. The solution was mixed with the PVP-pluronic solution followed by stirring at room temperature and the resulting precursor gel was heated at 50°C for 24 hrs. The gel was then electrospun and the formed fibers were calcined at 500°C for 4 hrs under air to form crystalline titanium dioxide nanofibers	[168]

Silver (Ag) compounds	In situ production of silver nanoparticles on cotton fabric is described and their antimicrobial potential is evaluated	Cotton fabric was introduced into a loading bath containing silver nitrate. To this solution CTAB and glucose were added and the mixture was shaken at 50°C. Subsequently, sodium hydroxide and water were added and the mixture was further shaken at 50°C. The coated samples were thoroughly rinsed with water and dried. The silver coated samples were washed with nonionic detergent (Triton X-100) and then the fabrics were dried	[169]

Copper oxide (CuO)	Copper oxide nanoparticles prepared by electrochemical reduction displayed excellent antibacterial activity against *Escherichia coli* and *Staphylococcus *strains	Copper oxide nanoparticles were prepared by electrochemical reduction, using an electrolysis cell in which a copper metal sheet served as a sacrificial anode and a platinum (inert) sheet acted as a cathode. For this process tetrabutylammonium bromide in an organic medium acted as a structure-directing agent which was used with acetonitrile (ACN) at a 4 : 1 ratio. The reduction process was allowed to takes place under an inert atmosphere of nitrogen for 2 hrs. Desired particle size was achieved by controlling parameters such as density, solvent polarity, distance between electrodes, and concentration of stabilizers	[170]

Iron oxide (Fe_3_O_4_) & zinc oxide (ZnO)	Zinc oxide was combined with iron oxide to produce magnetic composite nanoparticles with improved colloidal aqueous stability and adequate antibacterial activity	To prepare the Fe oxide nanoparticles, FeCl_2_·4H_2_O solution was added to a porcine gelatine aqueous solution, followed by addition of a NaNO_3_ solution and allowed to react for 10 min. Then the pH was raised to 9.5 by adding a NaOH aqueous solution (1 N). The Zn/Fe oxide composite nanoparticles were prepared similarly except for substituting the Fe^2+^ ions for a mixture of Fe^2+^ and Zn^2+^ of different weight ratios. The mixtures containing weight ratios [Zn]/[Fe] of 1 : 9, 3 : 7, 1 : 1, 8 : 2, and 9 : 1 were prepared by mixing different volumes of FeCl_2_·4H_2_O solution with the appropriate volumes of ZnCl_2_ solution. The procedure that followed was as described for the iron oxide nanoparticles	[171]

Magnesium oxide (MgO)	Magnesium oxide (MgO) nanowires (diameter, 6 nm; length, 10 *μ*m) were synthesized. These nanowires showed bacteriostatic activity against *Escherichia coli* and *Bacillus* species	A microwave hydrothermal technique was used to prepare MgO nanowires. In brief, an aqueous solution of a fixed concentration of urea was added dropwise to an aqueous magnesium acetate solution. The solution was then loaded into a microwave furnace. The product obtained was collected, dried, and calcined to obtain a white-colored final material	[172]

Nitric oxide (NO) nanoparticles	Nitric oxide- (NO-) releasing nanoparticle technology was used for the treatment of methicillin-resistant *Staphylococcus aureus *	First a hydrogel/glass composite was synthesized by adding tetramethyl orthosilicate, polyethylene glycol, chitosan, glucose, and sodium nitrite in sodium phosphate buffer. In this glass composite, nitrite was reduced to NO due to redox reactions initiated with thermally generated electrons from glucose. After the redox reaction, the ingredients were combined and dried using a lyophilizer, resulting in a fine powder consisting of nanoparticles containing NO. The water channels inside the particles of the hydrogel/glass composite opened in an aqueous environment, facilitating the release of the trapped NO over extended periods of time	[105]

Polyethylenimine and quaternary ammonium compounds	Antibacterial activity of quaternary ammonium polyethylenimine (PEI) nanoparticles embedded at 1% w/w in hybrid dental composite resins was determined	An ethanol solution of PEI was cross-linked with 8.7 mmol dibromopentane (PEI monomer/dibromopentane). The generated HBr was neutralized by treatment with sodium hydroxide and the resulting residue was purified from NaBr by gravitational filtration and dried under reduced pressure. The cross-linked PEI was further alkylated with bromooctane, as described above, to produce octane alkylated PEI. Octane alkylated PEI dispersed in anhydrous THF was reacted with methyl iodide in the presence of 2% cross-linked 4-vinylpyridine. The product was filtered to remove 4-vinylpyridinium salt and the filtrate was evaporated to dryness under reduced pressure	[173]

Chitosan & polyguanidines	Guanidinylated chitosan derivatives of different molecular weights were synthesized. Guanidinylated chitosan exhibited a fourfold lower inhibitory concentration compared with chitosan	A chitosan solution was prepared in HCl and then adjusted to pH 8-9 by 5% w/v aqueous sodium carbonate. The precipitate was washed with water and the desired amount of aminoiminomethanesulfonic acid was added. The reaction was kept at 50°C for 15 min and then the mixture was cooled to room temperature. Once cooled it was poured into saturated aqueous sodium sulfate, and the precipitate was filtered off, washed thoroughly with water and ethanol, and then dried under vacuum to give guanidinylated chitosan	[174]
